# The challenge of measuring physiological parameters during motor imagery engagement in patients after a stroke

**DOI:** 10.3389/fnins.2023.1225440

**Published:** 2023-07-31

**Authors:** Szabina Gäumann, Efe Anil Aksöz, Frank Behrendt, Jasmin Wandel, Letizia Cappelletti, Annika Krug, Daniel Mörder, Annika Bill, Katrin Parmar, Hans Ulrich Gerth, Leo H. Bonati, Corina Schuster-Amft

**Affiliations:** ^1^Department of Research, Reha Rheinfelden, Rheinfelden, Switzerland; ^2^School of Engineering and Information Technology, Bern University of Applied Sciences, Biel, Switzerland; ^3^Institute for Optimisation and Data Analysis, Bern University of Applied Sciences, Burgdorf, Switzerland; ^4^Department of Health Professions, Bern University of Applied Science, Bern, Switzerland; ^5^Institute for Physiotherapy, School of Health Professions, Zurich University of Applied Sciences, Winterthur, Switzerland; ^6^Department of Sport Science, Faculty of Humanities, University of Konstanz, Konstanz, Germany; ^7^Institute of Human Movement Sciences and Sport, Department of Health Sciences and Technology, ETH Zurich, Zurich, Switzerland; ^8^Department of Neurology, University Hospital Basel, Basel, Switzerland; ^9^Department of Medicine, University Hospital Münster, Münster, Germany; ^10^Department of Clinical Research, University of Basel, Basel, Switzerland; ^11^Department of Sport, Physical Activity, and Health, University of Basel, Basel, Switzerland

**Keywords:** motor imagery, stroke, electrooculography, validity, test–retest reliability, smart eyeglasses, pulse oximetry, electroencephalography

## Abstract

**Introduction:**

It is suggested that eye movement recordings could be used as an objective evaluation method of motor imagery (MI) engagement. Our investigation aimed to evaluate MI engagement in patients after stroke (PaS) compared with physical execution (PE) of a clinically relevant unilateral upper limb movement task of the patients' affected body side.

**Methods:**

In total, 21 PaS fulfilled the MI ability evaluation [Kinaesthetic and Visual Imagery Questionnaire (KVIQ-10), body rotation task (BRT), and mental chronometry task (MC)]. During the experiment, PaS moved a cup to distinct fields while wearing smart eyeglasses (SE) with electrooculography electrodes integrated into the nose pads and electrodes for conventional electrooculography (EOG). To verify MI engagement, heart rate (HR) and oxygen saturation (SpO_2_) were recorded, simultaneously with electroencephalography (EEG). Eye movements were recorded during MI, PE, and rest in two measurement sessions to compare the SE performance between conditions and SE's psychometric properties.

**Results:**

MI and PE correlation of SE signals varied between *r* = 0.12 and *r* = 0.76. Validity (cross-correlation with EOG signals) was calculated for MI (*r* = 0.53) and PE (*r* = 0.57). The SE showed moderate test–retest reliability (intraclass correlation coefficient) with *r* = 0.51 (95% CI 0.26–0.80) for MI and with *r* = 0.53 (95% CI 0.29 – 0.76) for PE. Event-related desynchronization and event-related synchronization changes of EEG showed a large variability. HR and SpO_2_ recordings showed similar values during MI and PE. The linear mixed model to examine HR and SpO_2_ between conditions (MI, PE, rest) revealed a significant difference in HR between rest and MI, and between rest and PE but not for SpO_2_. A Pearson correlation between MI ability assessments (KVIQ, BRT, MC) and physiological parameters showed no association between MI ability and HR and SpO_2_.

**Conclusion:**

The objective assessment of MI engagement in PaS remains challenging in clinical settings. However, HR was confirmed as a reliable parameter to assess MI engagement in PaS. Eye movements measured with the SE during MI did not resemble those during PE, which is presumably due to the demanding task. A re-evaluation with task adaptation is suggested.

## 1. Introduction

Imaging movements, also called motor imagery (MI), is a powerful mental training technique. In neurological rehabilitation, it has been shown to be an effective adjunct therapy to improve the motor learning process (Barclay et al., 2020). In a Cochrane review, Barclay and colleagues found that physical training combined with MI results in improved motor performance compared with physical training alone (Barclay et al., 2020). They described moderate-quality evidence that MI combined with physiotherapy or occupational therapy improves upper limb activity outcome (observed and self-perceived) and upper limb impairment outcome in patients after a stroke. The latest research showed more notable objective evidence of MI benefits if MI was used in conjunction with further modalities, e.g., action observation and vibration-based sensory stimulation (Lakshminarayanan et al., 2023a,b). MI is an attractive training method as it allows patients to practice various activities of daily living at any time without supervision and does not require special materials (Braun et al., 2013).

To ensure the efficacy of MI, it is necessary to determine whether a patient can engage in MI and, therefore, accurately perform MI tasks. Thus, it is suggested to assess the patients' MI ability prior to MI training (Dettmers et al., 2012; Schuster et al., 2012). For the clinical evaluation of MI ability, it is recommended to include psychometric and behavioral methods, e.g., self-reporting questionnaires and accuracy or temporal congruency tests (Di Rienzo et al., 2014). Furthermore, Di Rienzo et al. (2014) proposed to evaluate neurophysiological parameters; e.g., electromyography or functional magnetic resonance imaging provides an objective assessment of MI ability.

**Figure 1:**
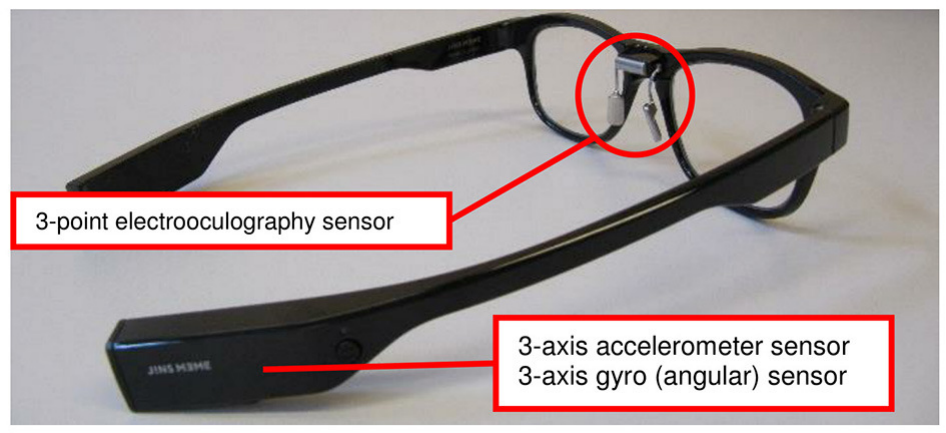
The J!NS MEME smart eyeglasses.

Oculomotor activity seems to be relevant in the mental simulation process and the creation of a mental image (Spivey and Geng, 2001; Heremans et al., 2008). In healthy individuals, Spivey and Geng (2001) found saccades in the same direction as the spatiotemporal dynamics of an auditory presented scene description, whereas Laeng et al. (2002) showed that the oculomotor activity pattern was similar during retrieving a visual information and during its imagery. Furthermore, de'Sperati (2003) revealed spontaneous ocular behavior during the imagination of a circular motion. Eye movements during MI and physical execution (PE) of a gesture showed further remarkable similarities in their characteristics. In healthy individuals, Heremans et al. (2008) described that the number and amplitude of eye movements during imagery resembled those made during PE of a goal-directed upper limb movement task (wrist flexion–extension). Therefore, the authors suggested that eye movement registration using electrooculography (EOG) provides an objective measurement option of MI ability (Heremans et al., 2008). EOG signal recordings can be used for MI ability assessment not only in healthy individuals but also in neurological patients (Heremans et al., 2013). Heremans et al. (2012b) found that patients with multiple sclerosis had longer eye movement time (duration between gaze fixation at the start and end of the movement task) compared with healthy controls during both MI and PE. The authors furthermore described the promoting effect of visual and auditory cues on the spatial accuracy of the imagined movement. In a further study, Heremans et al. (2012a) investigated EOG during MI and PE in patients with Parkinson's disease, focusing on the effect of external cueing. Patients were introduced to a task that was close to clinical routine, namely, the Box and Blocks Test. The authors described that patients with Parkinson's disease showed similar eye movement time and eye movement frequency during MI and PE. In a review, Heremans et al. (2013) concluded that eye movement recording is useful to assess MI ability in neurological patients. It is suitable mainly in research settings and could be an adequate instrument to evaluate MI ability in clinical routine (Heremans et al., 2012a, 2013). However, new wearable sensor technology could potentially provide a mobile solution to measure MI ability. The J!NS MEME smart eyeglasses (JIN CO., LTD., Japan) are equipped with three-point electrooculography electrodes, a three-axis accelerometer, and a gyroscope (Figure 1). The device measures wireless body axis movements, eye blinking, and eye movements in real time. Yet, to use smart eyeglasses as a wearable MI ability measurement device its psychometric properties must be evaluated. For patients, wearable, real-time measurement of MI ability during simple and complex activities of daily living would provide an objective MI ability assessment. This, in turn, would allow us to optimize and tailor MI training for inpatient and home training.

Therefore, the primary aim was to evaluate MI engagement in patients after stroke compared with the PE condition of a clinically meaningful unilateral upper limb movement task using EOG. We hypothesized that eye movements show large similarities during MI and PE. Furthermore, we aimed to assess the validity and the test–retest reliability of the J!NS MEME smart eyeglasses compared to conventional EOG to detect eye movements. We hypothesized that smart eyeglasses are a valid and reliable measurement device for the detection of eye movements during MI engagement in patients after a stroke.

Additionally, we aimed to show a similar activation of the central and autonomic nervous system during MI and PE of a unilateral upper limb goal-directed task in patients after stroke. Here, we intended to observe physiological responses in heart rate (HR) and oxygen saturation (SpO_2_), furthermore, event-related synchronization (ERS) and event-related desynchronization (ERD) measured in the primary sensorimotor area, thus verifying whether patients were engaged in MI.

## 2. Methods

### 2.1. Patient eligibility criteria

In- and outpatients after a stroke were recruited in a neurorehabilitation clinic in the Northwestern part of Switzerland between September 2019 and July 2021. Due to the COVID-19 pandemic, recruiting had to be suspended from March to August 2020. Potential study patients received oral and written information and had at least 24 h to consider participation. Data collection began after signing the study consent form. Patients were included in the study if they fulfilled the eligibility criteria listed in Table 1.

**Table 1:** Patient eligibility criteria.

**Inclusion criteria**	**Exclusion criteria**
• Age ≥ 18 years • First-ever clinically confirmed stroke • Able to sit stabile on a chair without armrests • Able to read and understand German • Able to see a fixation cross on a computer screen and a recognition mark on the table directly in front of patient without vision correction by eyeglasses • Able to perform a hand grasping and arm lifting task without external assistance	• Implanted cardiac pacemaker or implanted cardioverter-defibrillator • Presence of pain during assessment and measurement • Severe impairments in cognition and communication, which limits the study participation (Montreal Cognitive Assessments score ≤ 19) • Severe spatial–visual disorder • Additional neurological or psychiatric diseases • Peripheral facial nerve paresis • Fail to create a mental image (two or more out of three MI ability assessments scored unsatisfactorily: short version of the Kinaesthetic and Visual Imagery Questionnaire (KVIQ-10) score < 30, body rotation task < 75% of maximum score, mental chronometry ratio out of the range 1 ± 0.25)

### 2.2. Materials and procedures

The investigation was conducted in accordance with the Declaration of Helsinki and was approved by the local ethical committee (EKNZ no: 2019-00348, 2020-00545). Patients underwent two measurement sessions within 7 days at least 48 h apart.

Patients' MI ability was assessed using the short version of the Kinaesthetic and Visual Imagery Questionnaire (KVIQ-10), the body rotation task (BRT), and the mental chronometry (MC) task. These assessments evaluate distinct aspects of MI ability and are suggested to be applied in combination for an accurate assessment of individuals' MI ability (Di Rienzo et al., 2014). All of them are commonly applied for MI ability assessments in neurological patients (Heremans et al., 2013).

The KVIQ-10 is a reliable and valid instrument to evaluate the ability to visualize and feel the imagined movements in patients with sensorimotor impairments (Malouin et al., 2007; Schuster et al., 2012). Patients rate the clarity and sensation intensity of five imagined movements using a five-point rating scale: from 1 = no image to 5 = as clear as seeing, respectively, 1 = no sensation to 5 = as intense as executing the movement.

The BRT refers to an unconscious process, called implicit MI, and it determines individuals' ability to imagine moving their internal representation of their own body parts into a presented position or angle (Fiorio et al., 2006). Patients were required to determine the laterality of 32 pictures of a human hand or foot. The number of correct answers determined their score.

The MC examines the temporal coupling of MI (Malouin et al., 2008). To determine MC, the ratio of time needed to imagine and execute a grasping task was calculated.

To record eye movements on patients' affected body side, the EOG signals were recorded with a sampling frequency of 1000 Hz. After skin preparation, one pair of surface electrodes (BlueSensor N, Ambu, Germany) was placed on the vertical axis over the pupil. Data were captured using wireless sensors (Myon aktos classic, myon JSC, Switzerland) and the proEMG stand-alone software (V.2.1, Prophysics JSC, Switzerland). Simultaneously, patients wore the J!NS MEME smart eyeglasses (JIN CO., LTD, Japan, Figure 1). The smart eyeglasses are equipped with a 3-axis accelerometer, a 3-axis gyroscope sensor, and a 3-point electrooculography sensor integrated into the nose pads. The metal dry electrodes, two on both sides of the rhinion and one on the nasion, measured the electrical potential in μV, thus detecting horizontal and vertical eye movements and blinking frequency (Kanoh et al., 2015). Data acquisition took place with a sampling frequency of 100 Hz while the signal was transmitted in real time via Bluetooth connection using the J!NS MEME Data Logger software (V.1.1.10, JIN CO. LTD., Japan).

Cortical activity was measured using a 64-channel EEG cap (BE PLUS LTM Prewired Headcap, EBNeuro, Italy) and a wireless headbox with a sampling frequency of 1000 Hz (during the preprocessing, the frequency was down-sampled to 250 Hz). The position of the Ag/AgCl electrodes followed the 10–20 international system. Electrode impedances were kept below 20 kΩ, and data were acquired using Galileo NT Line software (2016, EBNeuro, Italy). For the ERD/ERS analysis, alpha (8–12 Hz) and beta (13–30 Hz) bands were evaluated.

Furthermore, oxygen saturation (SpO_2_) and heart rate (HR) were monitored and recorded continuously using a pulse oximeter that was included in the EEG recording device.

Recordings from the different measurement systems were synchronized using an external data acquisition system (LabJack U3-LV, LabJack Corporation, Lakewood, USA).

**Figure 2:**
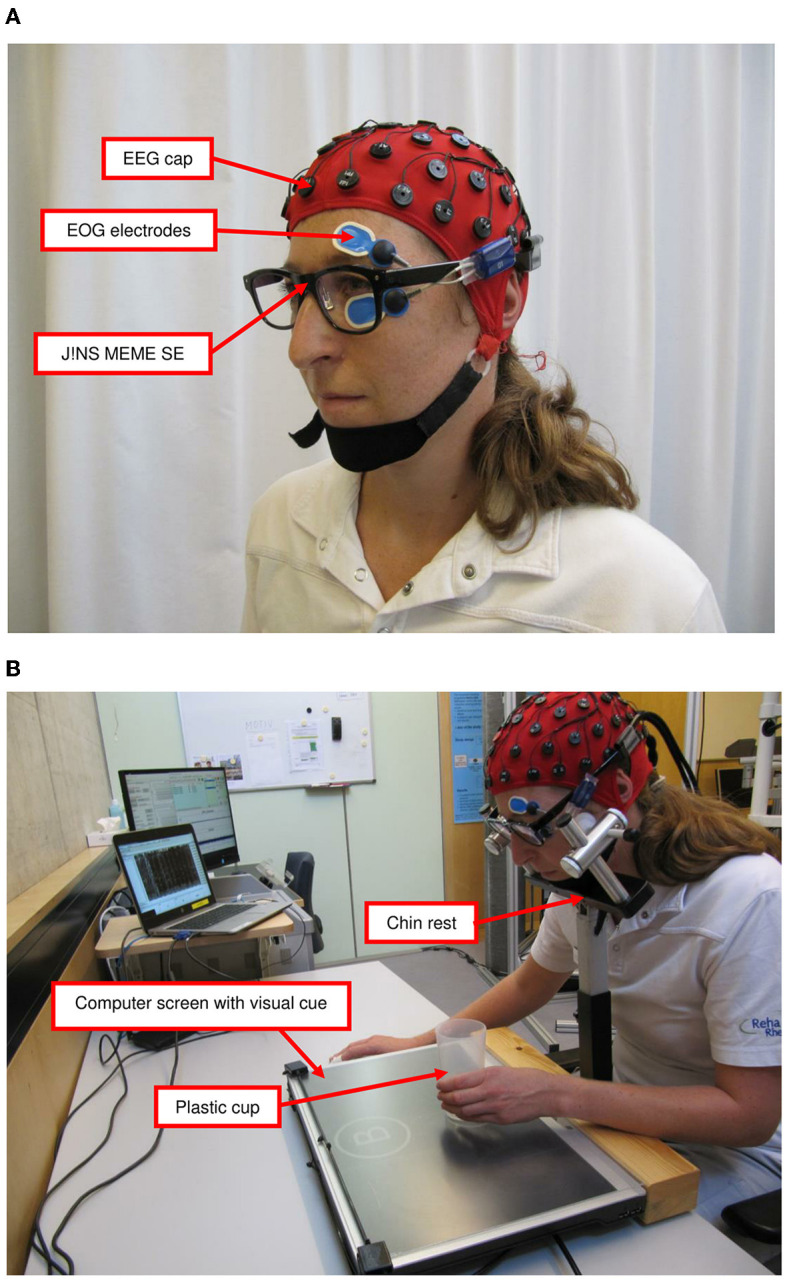
Measurement setting: **(A)** measurement devices and **(B)** measurement setting for upper limb task. EEG, electroencephalography; EOG, electrooculography; SE, smart eyeglasses.

Patients sat in front of a computer screen and received auditory and visual instructions for the tasks from the speakers of a connected laptop (Figures 2A, B). The task was designed using the PsychoPy software (3.0./2020.1.3., Open Science Tools Ltd., Great Britain) (Peirce et al., 2019). A customized chinrest (Gerald Kann—Kinnstützen und mechanische Vorrichtungen, Magdeburg, Germany) restricted head movements. The following standardized recording procedure was applied:

1) Resting phase: For 30 s, patients were asked to look at a fixation cross.
2) Familiarization phase: Patients were asked to look upward, downward, to the left and right sides according to a presented arrow on the screen without any head movements.
3) Task execution: Patients executed or imagined a unilateral upper limb movement task on their affected body side. In a random order, they imagined or executed placing a cup to marked fields A, B, or C (physical tasks: pA, pB, pC; imagery tasks: iA, iB, iC). Moving to A required a horizontal movement to the left side, moving to C required a horizontal movement to the right side, and moving to B required a vertical movement of the respective limb. This goal-directed movement task was chosen, as they require eye–hand coordination and we expected task-related eye movements based on the literature (Heremans et al., 2008, 2009).

**Figure 3:**
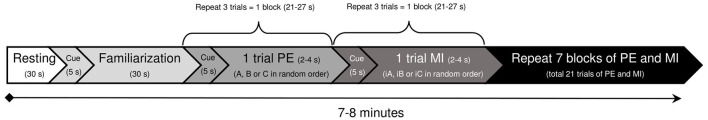
Experimental paradigm (one run). S, second; MI, motor imagery; PE, physical execution; A, B, or C, place a cup to marked fields A, B, or C; iA, iB, or iC, imagination of the task to marked fields A, B, or C.

During the complete recording procedure, patients were asked to keep their eyes open and were instructed to begin the task after the auditory and visual cues. They were encouraged to observe their movement during PE of the motor tasks. Each run compromised 21 trials of MI and PE of the task in blocks of three (Kobelt et al., 2018). A PE block was constantly followed by an MI block. A trial consisted of 5-s instruction and 2- to 4-s PE or MI period. There were two runs, which led to 42 physical and 42 imagery repetitions of the task. The patients had a rest between the runs. Thus, the MI and the PE data collection lasted about 6 min each (9 s × 21 trials × 2 runs). The experimental paradigm is shown in Figure 3.

An event start point was labeled precisely according to the recording log-files: the time point of the end of auditory and visual cues. During the experiment, the assessor monitored the exact termination of the physical and imagery task: if the cup was placed back to the starting point or if the patient indicated the end of the imagination by a tap with his/her non-involved hand. The assessor marked these ends as event points in the recording log-files by pressing a keyboard key.

### 2.3. Data analyses

Based on the literature, 14 to 20 participants are sufficient to evaluate psychometric properties and to recognize MI-related eye movements (Heremans et al., 2012a,b; Jang et al., 2018). Additionally, we performed a sample size calculation based on our EOG and SE data measured during the previously conducted technical validation of the setup and experimental task with five healthy individuals. Using the G^*^Power software (V.3.1, Heinrich Heine University Düsseldorf, Düsseldorf, Germany; Faul et al., 2007), a sample size of 40 was estimated with a power level of 0.8 and alpha level of 0.05 with an expected correlation *r* ≥ 0.60 between oculomotor activity during MI and PE. However, during the study implementation the COVID-19 pandemic forced us to reduce the number of included patients.

#### 2.3.1. Oculomotor activity compared during motor imagery and physical execution

Oculomotor activity recorded with the smart eyeglasses during PE was compared with oculomotor activity during MI using an amplitude-independent analysis, the cross-correlation for each patient and each task (pA/iA, pB/iB, pC/iC). The cross-correlation measures the similarity in shape between signals. The correlation value can range between 0 and 1. The more similar the shape between signals the higher the correlation value (Wren et al., 2006). A correlation *r* of ≥ 0.60 indicates excellent, r ranging between 0.31 and 0.59 adequate, and *r* of ≤ 30 a poor correlation. The calculations were performed using the xcorr function for the output values of the horizontal and vertical smart eyeglasses that were extracted according to their event name and normalized based on the shortest execution time using MATLAB (R2021a, MathWorks, Natick, USA).

#### 2.3.2. Psychometric properties of the smart eyeglasses

The validity of the SE compared with conventional EOG was calculated with the cross-correlation, for each patient and each condition. The conventional EOG signal was recorded with a sampling frequency of 1000 Hz and SE signal with a sampling frequency of 100 Hz. In order to make signals comparable, the conventional EOG data were down-sampled to 100 Hz. Mean EOG activity over all trials of each condition (MI, PE) was calculated on time-normalized raw EOG and smart eyeglasses data for each patient. Recordings of the first measurement session from 21 patients were analyzed. A correlation *r* of ≥ 0.60 is considered to indicate good convergent validity of the smart eyeglasses (Salter et al., 2005). The calculations were performed using the xcorr function with a lag range of 10 due to downsampling in MATLAB (R2021a, MathWorks, Natick, USA).

To determine the test–retest reliability of the smart eyeglasses, intraclass correlation coefficients (ICC, model: 2,1) with a 95% confidence interval (CI) were calculated for all patients and each condition (MI, PE) (Koo and Li, 2016). ICC values are considered to indicate reliability as follows: ICC of < 0.5 poor, between 0.5 and 0.75 moderate, between 0.75 and 0.9 good, and >0.9 excellent reliability (Koo and Li, 2016). Data from the first and the second measurement sessions were included in the analyses. A total of 18 patients completed both measurement sessions.

#### 2.3.3. Electroencephalography recordings

The preprocessing of raw EEG signals was carried out with a wrapper toolbox for MATLAB (Automagic; Pedroni et al., 2019). The toolbox, by default, uses the early-stage preprocessing pipeline (PREP pipeline) and adds further processing stages (Bigdely-Shamlo et al., 2015). The automated pre-processing pipeline for big datasets was demonstrated to be an efficient and reliable technique for both resting state and evoked EEG, which was evaluated in patients and healthy individuals (Da Cruz et al., 2018). The typical workflow of the Automagic toolbox was followed for pre-processing and is described in detail in the Supplementary Datasheet 1. ERD/ERS features in frequency bands, alpha (8–12 Hz) and beta (13–30 Hz), were extracted in different channel configurations (i.e., C1, C2, C3, CZ, C4, F3, F4, T7, T8, PZ) (Pfurtscheller et al., 2006; Li et al., 2015; Tabernig et al., 2016). The ERD/ERS values are defined as the proportional power change (ERD, power decrease, ERS, power increase) with respect to baseline activity within a given reference interval; 42 EEG signals of 5-s length (including 1-s baseline period before event onset) were obtained per patient for MI and PE of the grasping task. There was a particular interest in three regions: Cz (the center of the cerebral cortex), C3 (the left of the cerebral cortex), and C4 (the right of the cerebral cortex), which are recommended to be ideal for distinguishing MI situations in the literature (Hu et al., 2014). C3, C4, and Cz have been shown to be ideal for identifying MI states in EEG-based brain–computer interface studies (Pfurtscheller et al., 2006; Wang et al., 2007; Ge et al., 2014). The ERD/ERS calculation was carried out using the equations introduced by Graimann and Pfurtscheller (Graimann and Pfurtscheller, 2006) and is described in the Supplementary Datasheet 2.

The average ERD/ERS values were calculated for 12 datasets. The calculation was repeated for alpha and beta power bands and 10 electrode locations (C1, C2, C3, CZ, C4, F3, F4, T7, T8, PZ) for every 42 events (six events: pA, pB, pC, iA, iB, iC in a total of seven blocks). The average ERD/ERS values for event type (MI/PE) were used for statistical comparison. The analysis of variance (ANOVA) linear mixed-effects model was used (lme4, RStudio 2021.09.1 + 372). The model included time, event type (MI vs PE), age, time after stroke, KVIQ-10, BRT, and MC, and the Montreal Cognitive Assessment scores were taken as factors. To determine significance, a *p*-value of 0.05 was used as a threshold.

#### 2.3.4. Correlation of physiological measures and motor imagery ability

The EEG measurement device recorded the HR and SpO_2_ values too. Recordings from a total of 12 patients were involved in the analyses. The dataset was cut according to the timestamps taken from the recording log-file and assigned to the corresponding events: resting phase, PE of the task (pA, pB, pC), and MI of the task (iA, iB, iC). The means and standard deviations for each event were calculated.

The data of the MI ability assessments were tested for normal distribution with the Shapiro–Wilk test. Additionally, the measured HR and SpO_2_ values were checked for normal distribution with the Shapiro–Wilk test and potential outliers above the limit of 1.5^*^ of the interquartile range and are presented in QQ plots. All descriptive statistics were conducted with RStudio (Version 2022.7.1.554, RStudio Team, PBC, Boston, MA, USA).

To examine between-condition differences between MI, PE, and rest, the linear mixed model including confidence intervals (CI) was generated in MATLAB (2022) with the fitlme function (Pinheiro and Bates, 1996). We then followed the recommendations by Barr et al. (2013) to maximize the random effects in order to reduce type I errors (Barr et al., 2013). Thus, a linear mixed model was created with random intercepts and random slopes according to (1):


(1) $$\begin{array}{l} lme=fitlme\;\left(HR,{\;}^{{}^{\prime }}HR\;\sim \;condition\;+\;{\left(condition\;|\;id\right)}^{{}^{\prime }}\right) \end{array}$$

Here, HR stands for heart rate, the condition can be active, imagined, or resting and id reflects the patient. The same applies to oxygen saturation as the dependent variable.

To calculate the correlation between MI ability and physiological parameters, HR and SpO_2_ values (during MI) were averaged over the events and set in relation to the corresponding resting phases. Finally, the conditions were expressed as relative changes to the resting values, which is the standard procedure (Decety et al., 1991; Oishi et al., 2000). The correlations between the mean measurement changes and the assessment scores (KVIQ-10, BRT, MC) were calculated in RStudio Team (2022) using Pearson's product moment correlation. Significance was determined with *p*-value of < 0.05.

## 3. Results

**Figure 4:**
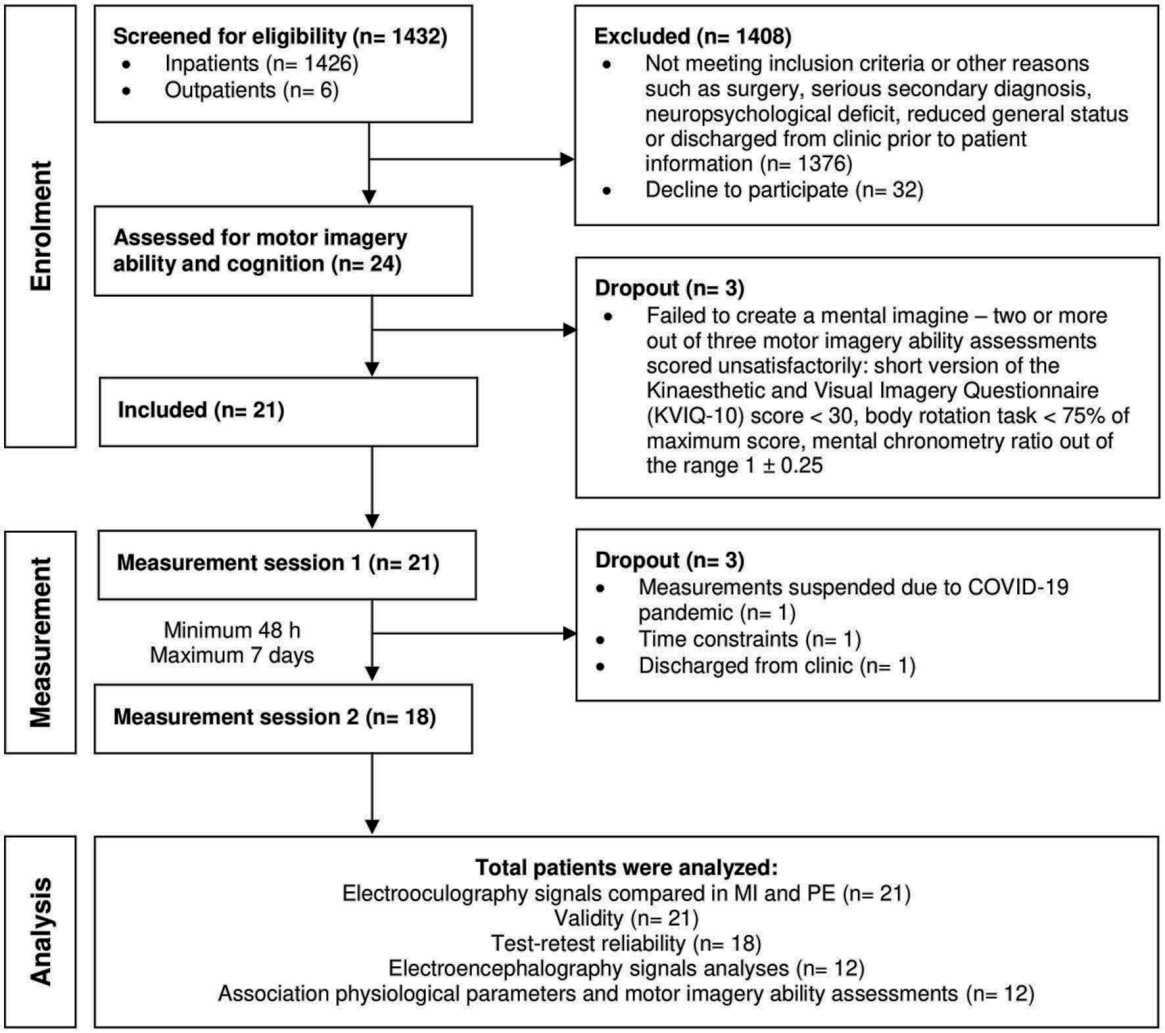
Patient flow chart. *n*, sample size.

In total, 21 patients participated in the study after giving written informed consent (see patient flow chart in Figure 4). Table 2 presents all patients' characteristics.

**Table 2:** Patients' characteristics (*n* = 21).

**Characteristic**	**Frequency or mean ±standard deviation**
Gender (female/ ale)	6/15
Age (years)	60.7 ± 15.9
Stroke (ischemic/hemorrhagic)	19/2
Time since diagnosis (days)	1108.6 ± 3508.4
Affected body side (left/right)	9/12
Edinburg handedness inventory (left dominant/right dominant)	2/19
Montreal cognitive assessment (score 0–30)	26.9 ± 2.2
Kinaesthetic and visual imagery questionnaire-10 (score 10–50)	39.1 ± 7.8
Body rotation task (score 0–32)	29.2 ± 2.7
Mental chronometry ratio (imagery/physical execution)	0.87 ± 0.2

### 3.1. Oculomotor activity compared during motor imagery and physical execution

The correlation of smart eyeglasses signals between MI and PE varied between *r* = 0.12 and *r* = 0.76. The cross-correlation analyses of smart eyeglasses signals between MI and PE revealed mean correlation value *r* = 0.2 for task pA/iA, mean correlation value *r* = 0.16 for task pB/iB, and mean correlation value *r* = 0.19 for task pC/iC.

### 3.2. Psychometric properties of the smart eyeglasses

The cross-correlation was calculated for each patient and each condition (PE, MI). Table 3 presents the range of the correlation values for each task (PE: pA, pB, pC; MI: iA, iB, iC). The highest estimate revealed the correlation during task pB with the value *r* = 0.71 and during task iB with the value *r* = 0.67 that described vertical eye movements. The mean correlation during PE overall (pA, pB, pC) was *r* = 0.57 and during MI overall (iA, iB, iC) *r* = 0.53.

**Table 3:** Results of validity and test–retest reliability analyses of the smart eyeglasses.

	**Validity**			**Test–retest reliability**			
	**Cross-correlation between SE and EOG signals (*****n** =* **21)**			**Intraclass correlation coefficients (*****n** =* **18)**			
**Event**	**Range**	**Mean**	**SD**	**Range**	**95%CI**	**Mean**	**SD**
pA	0.35–0.59	0.50	0.06	0.42–0.70	0.37–0.72	0.54	0.07
pB	0.59–0.79	0.71	0.06	0.33–0.68	0.27–0.71	0.52	0.10
pC	0.33–0.59	0.48	0.06	0.31–0.68	0.28–0.71	0.51	0.13
iA	0.31–0.60	0.47	0.08	0.33–0.70	0.29–0.70	0.51	0.10
iB	0.50–0.78	0.67	0.07	0.36–0.75	0.32–0.75	0.52	0.11
iC	0.31–0.60	0.45	0.08	0.29–0.71	0.24–0.72	0.52	0.13
Physical execution	0.22–0.67	**0.57**	0.12	0.32–0.73	0.29–0.76	**0.53**	0.10
Motor imagery	0.22–0.59	**0.53**	0.12	0.31–0.78	0.26–0.80	**0.51**	0.11

SE, smart eyeglasses; EOG, electrooculography; n, sample size, pA, pB, pC, physical execution of the task; iA, iB, iC, imagined execution of the task; SD, standard deviation; CI, confidence interval. The values in bold represent the overall mean values for physical execution and motor imagery, respectively.

The ICCs were calculated for each patient, each condition (PE, MI), and each task (PE: pA, pB, pC; MI: iA, iB, iC). Every task revealed similar ICCs ranging between 0.51 and 0.54. The average ICC was 0.53 (95% CI 0.29–0.76) for PE and 0.51 (95% CI 0.26–0.80) for MI. All ICCs with confidence intervals are listed in Table 3.

### 3.3. Statistical comparison of the ERD/ERS

**Figure 5:**
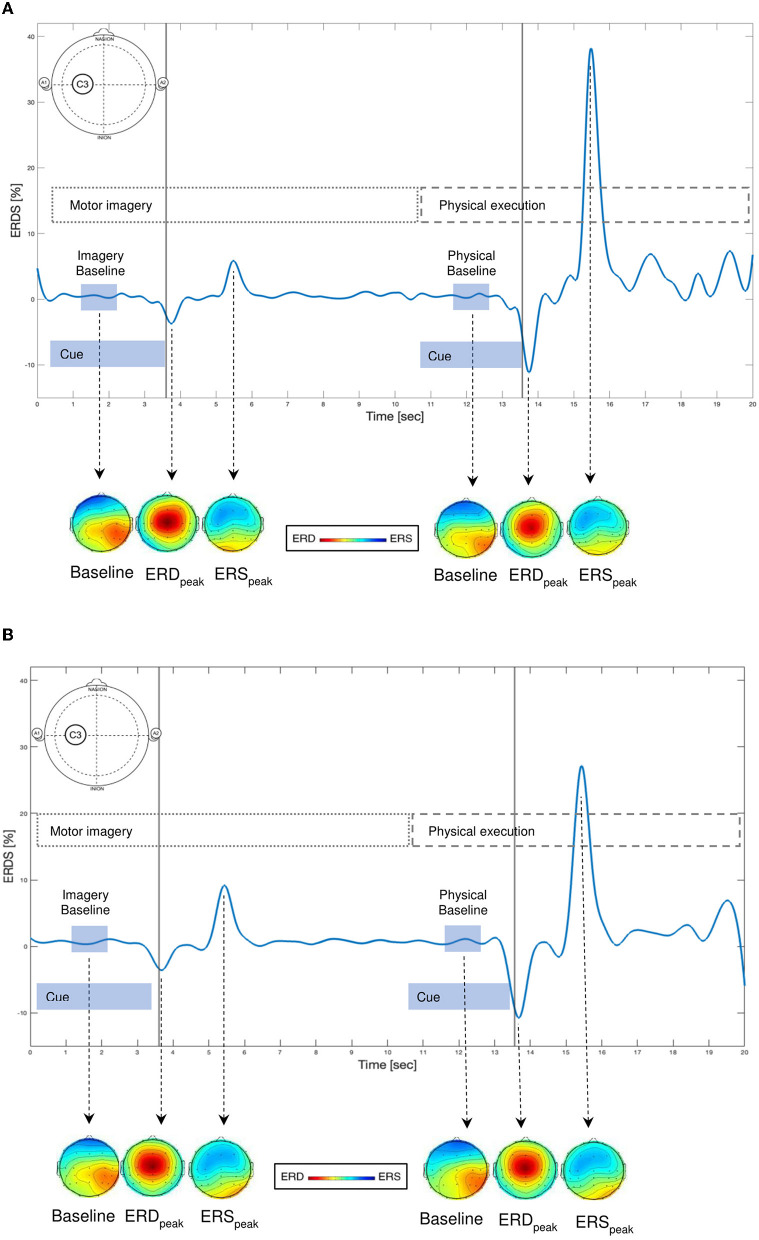
**(A, B)** Examples of time courses of ERD (event-related desynchronization, power decrease) and ERS (event-related synchronization, power increase) changes during imagery and physical execution of a grasping task for one patient (P009) and for electrode location C3 in the **(A)** alpha (8–12 Hz) and **(B)** beta (13–30 Hz) frequency bands. Scalp maps display the topographical distribution of band power at baseline (one second before task), at ERD peak, and at ERS peak for motor imagery and physical execution of the grasping task. Red indicates ERD and blue indicates ERS.

The time-frequency maps for electrode positions C3, Cz, and C4 showed large variability during different events. In individual cases, similar ERD and ERS patterns could be recognized by visual inspection during MI and PE. Figures 5A, B visualizes an example of band power 8–12 Hz (A), band power 13–30 HZ (B) time course displaying ERD and ERS during MI and PE of the grasping task for one patient (P009) and for electrode location C3. Average ERD/ERS values were compared for every patient in 10 different electrodes for MI and PE events using mixed-effect ANOVA. There were no statistically significant similarities between the events (MI/PE) except for event C in the F4 electrode in the beta power band (*p* = 0.0373). Additionally, in the alpha band, time and age showed a significant effect for the CZ electrode. This indicates that the older the patient more the similar the ERD/ERS values during MI and PE. As the experiment progressed, the ERD/ERS values became more similar during the MI and PE conditions. ERD/ERS values used for ANOVA were the average values during all physical (pA, pB, pC) and imagery (iA, iB, iC) events. The ERD/ERS values were then examined for each event separately and are displayed in Table 4.

**Table 4:** Confidence intervals for ERD/ERS values for alpha and beta power bands in 10 different channels.

**Electrode location**	**Alpha-Band**		**Beta-Band**	
	**Estimate (95 % CI)**	**SD**	**Estimate (95 % CI)**	**SD**
F3	0.434 (−0.392, 1.259)	0.424	0.479 (−0.115, 1.073)	0.306
F4	−0.536 (−1.298, 0.226)	0.393	−0.525 (−1.104, 0.054)	0.296
T7	1.148 (0.006, 2.290)	0.589	0.643 (−0.150, 1.435)	0.409
C3	−0.127 (−0.512, 0.257)	0.198	0.232 (−0.813, 1.277)	0.538
CZ	−0.319 (−1.735, 1.096)	0.725	0.208 (−0.759, 1.174)	0.496
C4	−0.343 (−0.978, 0.292)	0.327	0.561 (−0.330, 1.451)	0.459
T8	−0.416 (−1.492, 0.661)	0.555	−0.025 (−0.320, 0.270)	0.151
PZ	0.010 (−1.331, 1.352)	0.692	0.019 (−1.005, 1.043)	0.528
C1	0.443 (−0.610, 1.495)	0.542	−0.377 (−1.391, 0.637)	0.522
C2	0.030 (−1.017, 1.078)	0.540	−0.442 (−1.515, 0.632)	0.554

Estimate and SD columns present the mean of the ERDS values and standard deviations for each electrode.

### 3.4. Correlation of physiological measures and motor imagery ability

Descriptive statistics for the collected SpO_2_ values showed a normal distribution when tested with the Shapiro–Wilk test. The data showed no outliers in the referring range (1.5^*^ interquartile range). The HR datasets for all conditions followed a normal distribution determined with *p* > 0.05 in the Shapiro–Wilk test. The statistical description of the SpO_2_ and HR data collected for the upper limb task and the details for the distribution are presented in Table 5.

**Table 5:** Descriptive statistics for the peripheral oxygen saturation, heart rate, and motor imagery ability assessments.

***n =* 12**	**Mean**	**SEM**	**SD**	**Shapiro–Wilk**	***p-*value**	**Minimum**	**Maximum**
**Oxygen saturation**							
Physical execution	95.77	0.44	1.51	0.96	0.76	93.13	98.14
Mental execution	95.78	0.41	1.42	0.96	0.74	93.47	98.00
Rest condition	95.82	0.39	1.36	0.86	0.0502	92.88	97.08
**Heart rate**							
Physical execution	77.14	3.36	11.65	0.94	0.50	57.36	93.23
Mental execution	76.89	3.33	11.54	0.93	0.40	57.00	91.77
Rest condition	74.89	3.27	11.33	0.94	0.55	52.28	89.26
**Motor imagery ability**							
KVIQ-10	40.64	2.12	7.04	0.89	0.13	28	50
Body rotation task	29.55	0.72	2.39	0.89	0.14	24	32
Mental chronometry task	0.90	0.08	0.28	0.95	0.60	0.33	1.46

n, sample size; SEM, standard error of the mean; SD, standard deviation; KVIQ-10, short version of kinaesthetic and visual imagery questionnaire.

The results of the MI ability assessments were normally distributed as indicated by the Shapiro–Wilk test. The details of the descriptive analysis are shown in Table 5. The descriptive statistics (Table 5) for the different conditions showed no major differences in the SpO_2_ values but for HR.

The results of the linear mixed model with the corresponding estimates and the confidence intervals are presented in Table 6. According to the linear mixed model, no differences in the SpO_2_ values were identified between all experimental conditions. The statistical analysis of the applied linear mixed model for the HR values revealed a significant difference when the imagined condition was tested against the resting condition. Conversely, HR showed no significant difference from the imagined to the active condition. The complete analysis is presented in Table 6.

**Table 6:** Fixed-effects coefficients (95% CIs) for oxygen saturation and heart rate.

	**Estimate**	**SE**	**tStat**	**df**	***p-*value**	**Lower**	**Upper**
**Oxygen saturation**							
(Intercept)	95.78	0.40	242.45	33	3.1E-55	94.98	96.59
condition_rest	0.01	0.16	0.03	33	0.97	−0.31	0.32
condition_act	−0.02	0.05	−0.32	33	0.75	−0.13	0.09
**Heart rate**							
(Intercept)	76.89	3.19	24.12	33	1.56E-22	70.41	83.38
condition_rest	−2.00	0.44	−4.53	33	0.000073	−2.90	−1.10
condition_act	0.24	0.16	1.52	33	0.14	−0.08	0.57

The intercept corresponds to the mean estimate of the baseline factor levels. Here, the mean estimate of the oxygen saturation or the heart rate (as the dependent variables) for the imagined condition of the upper extremity. Other estimates were tested against the intercept. CI, confidence interval with lower/upper limits; SE, standard error; df, degrees of freedom.

The results of the correlation analysis for the SpO_2_ and HR are summarized in Table 7. No significant correlations were found between the changes in SpO_2_ during MI and the scores of MI ability assessments. No significant correlations were found between the changes in HR and the scores of MI ability assessments.

**Table 7:** Correlations for oxygen saturation and heart rate and motor imagery ability assessments.

	**Oxygen saturation**			**Heart rate**		
**Pearson**	**BRT – SpO**_2_	**MC – SpO**_2_	**KVIQ-10 – SpO**_2_	**BRT – HR**	**MC – HR**	**KVIQ-10 – HR**
*r*	−0.07	0.12	0.09	−0.05	0.39	0.39
*p*	0.83	0.72	0.78	0.88	0.22	0.21

BRT, body rotation task; MC, mental chronometry; KVIQ-10, short version of Kinaesthetic and Visual Imagery Questionnaire; SpO_2_, oxygen saturation; HR, heart rate.

## 4. Discussion

The present study aimed to evaluate MI engagement in patients after stroke using EOG electrodes integrated into the smart eyeglasses and compare oculomotor activity during PE and MI of a clinically relevant upper limb task. Our results showed a poor correlation of oculomotor activity measured with the smart eyeglasses during MI and PE. Furthermore, the validity and test–retest reliability of the J!NS MEME smart eyeglasses were evaluated compared with conventional EOG. Based on the results, we can conclude that the J!NS MEME smart eyeglasses showed an adequate validity and a moderate test–retest reliability.

A secondary aim of our study was to describe the neurophysiological changes in the primary sensorimotor area during MI and PE using EEG recordings. In individual cases, ERD/ERS changes were comparable to previously described and we could recognize MI- and PE-related patterns in EEG recordings (Pfurtscheller and Neuper, 1997, 2001). However, our findings showed that ERD/ERS changes are variable and there were no significant similarities when comparing MI and PE. Additionally, we aimed to investigate whether MI is reflected in physiological responses of the autonomic nervous system (ANS) under different conditions: MI, PE, and rest. The HR during MI and PE was found to be significantly different compared with the resting condition. Conversely, the imagined condition showed no significant difference from the active condition. Furthermore, we investigated the relationship between MI ability and physiological parameters, e.g., heart rate and oxygen saturation. There was no significant correlation between the physiological parameters such as HR and SpO_2_ during MI and the scores of the MI ability assessments.

### 4.1. Oculomotor activity compared during motor imagery and physical execution

Oculomotor activity measured with smart eyeglasses during MI demonstrated a low correlation with those during PE. Here, the recording frequency could have an impact on the results. Eye movements are usually recorded with a sampling frequency of 1024 Hz that allows to record fine movements (Heremans et al., 2008, 2009, 2011). The recording frequency of the SE in our study was predefined to 100 Hz only, which might not have been sufficient for recognizing MI. A further explanation could be the different strategies patients used to complete the task. In our experiment, it was not possible to directly control how precisely patients imagined the movement. Patients might have used a different strategy to complete the task, and eye movements were superfluous or they rather have used peripheral vision. To support this explanation, Heremans and colleagues also reported that 11 to 17 % of their healthy participants did not always show task-related eye movements (Heremans et al., 2008, 2009). Moreover, our patients used their self-selected speed to execute or imagine a movement, which could have resulted in an additional source of variability of eye movement patterns. This explanation is supported by the findings from Heremans et al. (2009), who stated that external auditory cues offered by a metronome enhance temporal accuracy.

However, our selected task itself could be taken into account. Studies investigating eye movements with EOG usually demand participants to carry out a strictly directed task. They apply visual or/and auditory cues (e.g., using a metronome to instruct the start of a movement and visualizing the start and end point of a task with a symbol), thus influencing duration and spatial accuracy (Heremans et al., 2008, 2012a,b; Lanata et al., 2020). In contrast, in our study, patients were free to choose how to perform tasks. We could conclude that the experimental task selected for an MI ability assessment requires more external control or cueing and standardization.

Additionally, studies describing large similarities in eye movement during MI and PE examined different parameters, which could explain the differences in our results. They analyzed eye movement amplitude (corresponding distance), number of eye movements, duration, or saccades (Spivey and Geng, 2001; Laeng et al., 2002; de'Sperati, 2003; Heremans et al., 2013) while we compared the shape of the EOG signal.

### 4.2. Psychometric properties of the smart eyeglasses

The validity of the smart eyeglasses was examined using a cross-correlation. The overall mean correlation value between the signals of the smart eyeglasses and conventional EOG during PE was *r* = 0.57 and during MI *r* = 0.53. According to the correlation classification for outcome measures in stroke rehabilitation of Salter et al. (2005), our findings imply an adequate cross-correlation. However, during physical task pB and imagery task iB the smart eyeglasses revealed a correlation value of *r* = 0.71 and *r* = 0.67 that fulfilled the desirable value r of ≥ 0.60 for excellent correlation (Salter et al., 2005). The task pB and iB involved mainly vertical eye movement. The higher values during these tasks can be explained by the electrode placement. Previous studies used four surface electrodes for conventional EOG (Heremans et al., 2008, 2009, 2011). In the current investigation, only one pair of electrodes was placed above and under the eye in a vertical line; consequently, more activity could be recorded when the eye was moving vertically. Another pair of electrodes were waived due to practical reasons. As EOG was simultaneously recorded with the smart eyeglasses and the surface electrodes, the smart eyeglasses occupied the nose and made the attachment of an additional pair of surface electrodes impossible. The head and face were already heavily covered with the smart eyeglasses and the EEG cap.

In addition, the recording frequency could have had an impact on the results. However, our conventional EOG was recorded with a sampling frequency of 1000 Hz. For comparison with the smart eyeglasses data EOG signals had to be down-sampled, which entails a great loss of accuracy thus affecting the correlation results. Yet, Barbara and Camilleri (2016) presented a blink accuracy of 97.6% and a saccade accuracy of 73.4% of the smart eyeglasses and these results were comparable to those obtained using surface electrodes. However, the authors examined the eye movements of healthy volunteers, and their findings provide a hint that the acquisition frequency of the smart eyeglasses might be adequate to capture eye movements. Furthermore, in the study of Barbara and Camilleri (2016), study participants were instructed to look at certain positions on a computer screen, while in our study, patients were not explicitly guided where to look.

The J!NS MEME smart eyeglasses showed moderate test–retest reliability to detect eye movements during PE and MI of a goal-directed upper limb motor task with overall ICC values of 0.53 (95% CI 0.29–0.76) for PE and 0.51 (95% CI 0.26–0.80) for MI (Koo and Li, 2016). Here, the explanation for the relatively low values could be the different strategies patients used for task execution. Furthermore, spontaneous eye movement patterns must have been considered as they could have overlapped task-related eye activity and therefore influenced our results. Both voluntary goal-directed (specific movement of the eye) and involuntary (blinking) eye movements were captured at the same time. Blinking frequency can vary according to daytime, fatigue, and concentration level (Wu et al., 2014; Kanematsu et al., 2019; Howell et al., 2020). In the present study, we could not ensure that both measurements were conducted at the same time of the day. In addition, our patients might have received other therapy sessions before the measurement, which could have further influenced their tiredness level and therefore their eye movements.

In accordance with the present results, previous studies have demonstrated similar reliability values. Howell et al. (2020) reported moderate test–retest reliability (r values ranged between 0.4 and 0.6) of eye-tracking assessments, and Skinner et al. (2018) presented similarly moderate reliability values (ICC −0.31 to 0.71) of eye-tracking with the Eyelink 1000 Eyetracker System (SR Research Ltd., Ottawa, Canada).

### 4.3. ERD/ERS during motor imagery and physical execution

Our findings showed a large variability of ERD/ERS changes during MI and PE of a grasping task. MI- or PE-related ERD/ERS were found in individual cases; however, not in all patients MI or PE related ERD/ERS could be described. Hence, ERD/ERS changes were not comparable with earlier findings and there were no significant similarities between ERD/ERS values during MI and PE (Beisteiner et al., 1995; Pfurtscheller and Neuper, 1997; Cremades and Pease, 2007; Nam et al., 2011; Tabernig et al., 2016; Wriessnegger et al., 2018; Daeglau et al., 2020). In one electrode location (F4), the ERD/ERS values during MI and PE became more similar over time. The reason could be that once the movement sequence is learned it is performed more automatically. A possible way to handle large variability of EEG signals during MI could be the use of classification algorithms that are mostly used in studies with a brain–computer interface to improve real-time processing (Ma et al., 2022).

In the present study, task duration was not predetermined. The duration changed depending on the patients as they were not instructed how long they should maintain imagery or perform the physical task; thus, the time elapse varied. We selected a 4-s epoch that should be sufficient to differentiate different imagery tasks (Neuper et al., 2006). The defined 4-s epoch was analyzed in every patient and for each task. Average ERD/ERS values were calculated in the fixed time window. The fixed interval could have influenced ERS/ERD variability as well.

Furthermore, an automatic baseline selection was introduced for each task (1 s later than the audio cue started), compared with other methods, when taking an individual baseline during every single trial (Pfurtscheller et al., 2006). Additionally, there were no breaks between individual tasks. That could result in an overlap in the EEG signals regarding the different tasks patients were supposed to carry out such as listening and understanding the audio cue, processing visual cues, as well as the preparation for mental or physical execution of the tasks. The ERS/ERD measurements could be improved by adapting the applied paradigm and introducing longer inter-event intervals (Pfurtscheller and Lopes da Silva, 1999). In addition, the introduction of longer task sequences (controlled duration) might improve data quality. However, brief imagery can result in a larger power decrease and thus might be easier to detect (Nam et al., 2011).

Kind of movement and imagery tasks and their influence on study results could be considered. Compared with other investigations in EEG changes during MI when a simple single joint movement (e.g., finger tipping) was examined, in the present study, a complex movement of daily living was explored. The coordination of a complex task might involve more cognitive processes resulting in more EEG activation signals.

Individual differences in imagery-related ERD/ERS changes were already described in the literature (Annett, 1995; Curran and Stokes, 2003; Solodkin et al., 2004). If there is no specific instruction on how to engage in MI, patients might use different MI perspectives (internal, external) and modalities (visual, kinesthetic) requiring different brain activation patterns or strategies. In the current study, patients were free to choose their MI varieties. Here, the control of MI parameters might improve inter-subject similarities.

### 4.4. Correlation of physiological measures and motor imagery ability

The ANS can provide valid and reliable measurements of MI (Collet et al., 2013). Therefore, we hypothesized that both physiological parameters HR and SpO_2_ would change depending on the condition. Our linear mixed model revealed HR to be significantly different between the imagined condition compared with the resting condition and between the active and the resting conditions. Furthermore, the imagined condition showed no significant difference to the active condition and both provoked very similar responses. These findings could imply that the active and imagined conditions underlie the same cognitive pathways that are reflected in the ANS response.

However, the measured SpO_2_ showed no significant difference for any of the conditions and remained constant during all three conditions. A possible explanation could be that SpO_2_ remains unaffected for low-intensity demands. Even for small responses, higher loads are necessary (Daglioglu et al., 2013). Here, our motor task might not have been demanding enough, to evoke a distinct reaction.

For the present study, HR was successfully identified as a physiological readout for MI performance but not SpO_2_. These results are in accordance with similar studies from Oishi et al. (2000) and Papadelis et al. (2007), who investigated ANS responses in healthy individuals. Further research should focus on MI tasks that are more demanding and hence provoke a stronger response. When the cardiovascular system is of interest, the more advanced and sensitive method of heart rate variability should be considered as a measurement parameter. It contains both the classical heart rate and its variability, which could provide a more comprehensive readout.

### 4.5. Study strengths and limitations

Prior to the present patient study, a technical validation of the setup and the experimental motor task were carried out that allowed to adjust the experiment to the needs of patients after stroke. Previous studies described detection of the eye movement in simple one-dimensional, e.g., wrist flexion and extension movements only (Heremans et al., 2008) compared with our study that included patients, who completed a complex activity of daily living tasks. Furthermore, most of the studies investigating eye movements during MI included healthy participants only. To our knowledge, the present investigation is the first study that involved patients after a stroke and evaluated their eye movements during MI compared with PE. Furthermore, our patients could select their preferred movement speed in both conditions MI and PE; thus, the spontaneous eye movements could be recorded in comparison with other studies with a strictly predetermined protocol (Heremans et al., 2012a,b).

The strength of the study is the numerous repetitions of the task in both conditions that resulted in sufficient data for the EEG analyses. Patients had to repeat each task 42 times, as it was recommended in previous investigations (Neuper et al., 2006; Pfurtscheller et al., 2006). Furthermore, an object-directed MI task (grasp and replace a cup) was used that could lead to a larger cortical activation (Li et al., 2015). Our EEG data pre-processing and analyses followed standardized procedures based on recommendations in the literature (Pedroni et al., 2019).

A limitation must be considered when interpreting the EEG findings. The MI and PE tasks might have been too short and could have led to an overlap of the activation of different brain regions. Thus, results have to be interpreted with caution.

In the present study, patients' medication was not recorded. However, the measurements took place within 7 days and it can be assumed that medication did not change in this short time period.

As MI is a mental simulation process, no external monitoring exists to control MI engagement and performance. In the present study, an attempt was made and received already promising results for the physiological parameters of HR. To our knowledge, it was the first clinical study that investigated HR to control for MI engagement in patients after a stroke.

## 5. Conclusion

The measurement of eye movements with EOG could provide an objective technique to evaluate MI ability in clinical routine and thus would allow us to optimize and tailor MI training. In the present study, we investigated eye movements during MI and PE of clinically meaningful goal-directed upper limb tasks in patients after a stroke. Eye movements measured with EOG electrodes integrated into smart eyeglasses during MI did not appear to be similar for PE. It remains unclear whether a motor task of activity of daily living is suitable for MI ability assessment using EOG and whether smart eyeglasses are suitable to differentiate MI and PE in a clinical setting. However, the J!NS MEME smart eyeglasses showed an adequate validity and a moderate test–retest reliability to detect eye movements during MI and PE of a grasping task.

With the analyses of EEG recordings and ANS responses, we attempt to verify MI engagement in patients after stroke. The current investigation demonstrated that there was large variability of ERD/ERS changes during MI and PE of a grasping task. MI- or PE-related ERD/ERS could be described in single cases. These results should be interpreted cautiously as the EEG pre-processing and the experimental paradigm could affect the results. No significant correlations were found between the MI ability and the physiological responses of HR and SpO_2_ during MI. Nevertheless, a significant difference in the HR between the three conditions (active, imagined, resting) was determined. HR seems to be a reliable indicator of the presence of MI.

In conclusion, the study provided a comprehensive evaluation of various physiological parameters during MI engagement in patients after stroke. Our results support the selection of adequate measurement methods and measurement parameters for future clinical MI investigations with patients in a clinical environment.

## Data availability statement

The raw data supporting the conclusions of this article will be made available by the authors, without undue reservation.

## Ethics statement

The studies involving human participants were reviewed and approved by the Ethikkommission Nordwest- und Zentralschweiz. The patients/participants provided their written informed consent to participate in this study. Written informed consent was obtained from the individual(s) for the publication of any potentially identifiable images or data included in this article.

## Author contributions

SG, EA, LC, AK, and CS-A designed the study. SG, LC, AK, and DM acquired data. SG, EA, JW, LC, AK, DM, and AB performed the data analysis. SG wrote the first draft of the manuscript. EA, JW, LC, AK, DM, and AB wrote sections of the manuscript. SG, EA, FB, and CS-A contributed to manuscript revision. All authors read and approved the final manuscript, were responsible for interpretation, and manuscript review.

## Supplementary material

Supplementary Datasheet 1 EEG preprocessing steps.

Supplementary Datasheet 2 Equations used for ERD/ERS calculation.
