# Supplementary material for: The challenge of measuring physiological parameters during motor imagery engagement in patients after a stroke
Source: Front Neurosci. 2023 Jul 31;17:1225440. doi: 10.3389/fnins.2023.1225440 (PMC10423937; doi:10.3389/fnins.2023.1225440)
Supplement: Supplementary Datasheet 2 — Equations used for ERD/ERS calculation. [file Data_Sheet_2.pdf]

Supplementary Material

The challenge of measuring physiological parameters during motor imagery in patients after stroke

Szabina Gäumann*, Efe Anil Aksöz, Frank Behrendt, Jasmin Wandel, Letizia Cappelletti, Annika Krug, Daniel Mörder, Annika Bill, Katrin Parmar, Hans Ulrich Gerth, Leo H. Bonati, Corina Schuster-Amft

*** Correspondence:** Szabina Gäumann: s.gaeumann@reha-rhf.ch

1 Supplementary Data

Pre-processing of EEG signals using a typical workflow of the Automagic toolbox for MATLAB (Pedroni et al., 2019):

a) The raw EEG data was first added to a project by using the graphical user interface of the Automagic toolbox. Each dataset inside a project is processed by the PREP pipeline.

b) Temporary (not affecting final EEG) data was detrended at 1.00 Hz cutoff (0.02s window slide) using high pass (pop_eegfiltnew FIR filter) detrending to improve the performance of stream processing.

c) Line noise removal were carried out by using routines from the cleanline EEGLAB plugin (Mullen, 2012) with a frequency range of [0.00 128.00] Hz, where 50-100 Hz (+/- 2 Hz bandwidth) were removed in 10 iterations. Noisy or outlier channels were detected based on the preprocessing pipeline.

d) After identifying bad channels, EEG data was high pass filtered using pop_eegfiltnew() FIR filter with passband edge(s): 0.10Hz, filter order: 8448.00, cutoff_freq: 0.05, transition band width: 0.10Hz. A low pass filter was performed using pop_eegfiltnew() FIR filter with passband edge(s): 30.00Hz, filter order: 114.00, cutoff_freq: 33.75, transition band width: 7.41Hz. A total of 5 noisy or outlier channels were removed.

e) Using linear analysis, the effect of EOG was also removed (Parra et al., 2005). No TrimOutlier was performed.

f) Automatic classification and removal of artifactual source components was carried out using the Multiple Artifact Rejection Algorithm (MARA) (Winkler et al., 2011).

g) Then, a temporary (not affecting final data) high pass filter was performed using pop_eegfiltnew() FIR filter with passband edge(s): 2.00Hz, filter order: 424.00, transition band width: 1.99Hz. An ICA decomposition of an EEG dataset was carried out using the EEGLAB function runica().

h) Finally, the quality of each dataset was evaluated using a variety of objective criteria and categorized as "Good", "OK" or "Bad" by applying cut-offs to the quality criteria. The dataset categorized as "Good" and "OK" were then used for further analysis.

2 Supplementary Data

ERD/ERS calculation using the equations introduced by Graimann and Pfurtscheller (2006) as seen in (1):

$$Act_j = \frac{1}{N}\sum_{i=1}^{N} {y_{ij}}^2$$

$$R = \frac{1}{k+1}\sum_{j=r_0}^{r_0+k} Act_{(j)}$$

$$ERDS_j(\%) = \frac{Act_{(j)} - R}{R} \times 100\%$$

(1)

where *N* is the total number of physical and imagery trials and y denotes the *j*th sample from the *i*th trial of filtered data. *Act* is the average power squared at the *j*th sample. The average reference interval power [ro, ro + k] is denoted by R. Finally, ERDS denotes ERD/ERS values of the *j*th sample in percentage.

References

Graimann, B., and Pfurtscheller, G. (2006). Quantification and visualization of event-related changes in oscillatory brain activity in the time-frequency domain. *Prog Brain Res* 159**,** 79-97. doi: 10.1016/s0079-6123(06)59006-5.

Mullen, T. (2012). CleanLine EEGLAB plugin. *San Diego, CA: Neuroimaging Informatics Toolsand Resources Clearinghouse (NITRC).*

Parra, L.C., Spence, C.D., Gerson, A.D., and Sajda, P. (2005). Recipes for the linear analysis of EEG. *Neuroimage* 28(2)**,** 326-341. doi: 10.1016/j.neuroimage.2005.05.032.

Pedroni, A., Bahreini, A., and Langer, N. (2019). Automagic: Standardized preprocessing of big EEG data. *Neuroimage* 200**,** 460-473. doi: 10.1016/j.neuroimage.2019.06.046.

Winkler, I., Haufe, S., and Tangermann, M. (2011). Automatic Classification of Artifactual ICA-Components for Artifact Removal in EEG Signals. *Behavioral and Brain Functions* 7(1)**,** 30. doi: 10.1186/1744-9081-7-30.
